# Uniting for Connection: Philanthropy’s Role in Coalition-Building and Advocacy Efforts

**DOI:** 10.1093/geroni/igaf122.1045

**Published:** 2025-12-31

**Authors:** Amy Eisenstein

**Affiliations:** RRF Foundation for Aging, Chicago, Illinois, United States

## Abstract

As social isolation and loneliness gain increasing recognition as urgent public health and societal challenges, philanthropy has a unique and powerful role in driving change. This discussion will explore how philanthropic organizations can strategically invest in coalition-building initiatives that advance social connection and scale evidence-based interventions. Specifically, it will focus on an example of current advocacy efforts to secure reauthorization of the Older Americans Act. Attendees will leave with a deeper understanding of how philanthropy can catalyze action and amplify impact in fostering social connection among the aging population.

